# Increasing trends of malaria in a border area of the Greater Mekong Subregion

**DOI:** 10.1186/s12936-019-2924-6

**Published:** 2019-09-12

**Authors:** Jinting Geng, Pallavi Malla, Jiaqi Zhang, Shiling Xu, Cuiying Li, Yan Zhao, Qinghui Wang, Myat Phone Kyaw, Yaming Cao, Zhaoqing Yang, Liwang Cui

**Affiliations:** 1 Department of Pathogen Biology and Immunology, Kunming Medical University, Kunming, China; 2 Division of Infectious Diseases and International Medicine, Department of Internal Medicine, Morsani College of Medicine, University of South Florida, 3720 Spectrum Boulevard, Suite 304, Tampa, FL 33612 USA; 3 Department of Immunology, College of Basic Medical Sciences, China Medical University, Shenyang, 110122 Liaoning China; 4 Myanmar Medical Association, Yangon, Myanmar

**Keywords:** Malaria, Elimination, China–Myanmar border, *P. vivax*, Risk factors

## Abstract

**Background:**

Intensive malaria transmission along international borders is a significant impediment to malaria elimination in the Greater Mekong Subregion (GMS) of Southeast Asia. Passive case detection (PCD) was used to study the dynamics and trends of malaria transmission at the China–Myanmar border to provide epidemiologic information for improved malaria control.

**Methods:**

PCD was conducted in one hospital and 12 clinics near the Laiza town in northeast Myanmar from 2011 to 2016. Clinical malaria was diagnosed by microscopy and demographic information was captured using a structured questionnaire at the time of the patient’s presentation for care.

**Results:**

Over the study period, 6175 (19.7%) malaria cases were confirmed by microscopy from 31,326 suspected cases. The four human malaria parasite species were all identified, with *Plasmodium vivax* and *Plasmodium falciparum* accounting for 5607 (90.8%) and 481 (7.8%) of the confirmed cases, respectively. In contrast to the steady decline of malaria in the general GMS, the study site had an upward trend of malaria incidence with vivax malaria outbreaks in 2013 and 2016. Adult males, children under the age of 15, and those with occupations such as farming, being a soldier or student, had significantly higher risks of clinical malaria compared to having fevers from other aetiologies. A self-reported history of clinical malaria was also associated with a higher risk of confirmed malaria.

**Conclusions:**

The China–Myanmar border area has experienced an overall upward trend of malaria incidence in recent years with *P. vivax* becoming the predominant species. Evidence-based control strategies need to focus on high-risk populations.

## Background

Between 2000 and 2015, malaria incidence declined from an estimated 237 million cases to 211 million cases and mortality decreased by 29% globally. However, these reductions appear to have plateaued, with 2016 and 2017 reporting a slight increase in the number of malaria cases [1]. The emergence and spread of parasites resistant to anti-malarial drugs and mosquitoes resistant to insecticides significantly hamper the global efforts to control and eliminate malaria [1]. In addition, reduction in financial support in high-burden countries was also responsible for the slight rebound of the global malaria incidence, despite overall relatively stable funding for malaria. The six countries of the Greater Mekong Subregion (GMS) of Southeast Asia have targeted malaria elimination by 2030 [2]. A major challenge to achieving this goal in the region is the cross-border movement of people from intensive malaria transmission areas along international borders. In the border regions, public health infrastructure and accessibility are poor, and people often live in extreme poverty [3]. Furthermore, civil unrests have led to internal displacement of human populations and movement of human populations to border areas from interior malaria-endemic areas, which further exacerbates the malaria problem along the borders [4, 5]. Among the GMS nations, the malaria burden and progress towards malaria elimination differs drastically by country. Myanmar had the heaviest malaria burden in 2016 [6], whereas China reported no autochthonous malaria in 2017 [1]. All reported cases in China were imported from endemic areas outside its borders. Thus, in this elimination phase, close surveillance at malaria transmission hotspots along international borders is critical to prevent cross-border re-introduction of the malaria parasites [7].

To track the progress of malaria control and elimination at the China–Myanmar border, surveillance using passive case detection (PCD) among febrile patients was implemented at treatment facilities in the Laiza Township, Kachin State, Myanmar.

## Methods

### Study site and populations

This study reports surveillance data collected between 2011 and 2016 in the Laiza Township (24°45′N, 97°33′E, altitude 263 m), Kachin State, Myanmar. The Laiza Township borders the Nabang Township located in the Yingjiang County of Yunnan Province, China. Laiza has a total population of ~ 20,000 based on the 2012 census. In 2010, as a result of military conflicts between local and central Myanmar governments, a few settlements for internally displaced people (IDP) established themselves at sites ~ 5 km from the Laiza town. Malaria case information was collected through PCD instituted in one hospital and 12 clinics in and around Laiza.

### Study design and data collection

Patient enrollment, data, and sample collection were performed by trained nurses. Febrile patients (axillary temperature ≥ 37.5 °C) or patients suspected of having malaria, with a history of fever within the last 24 h, were recruited into the study. Written informed consent, or assent in the case of minors, was obtained from all participants, prior to enrollment into the study. Demographic (ethnicity, age, gender, occupation, education, travel history) and clinical (fever, fever history, symptoms, malaria history, previous treatment) data were obtained using a structured questionnaire. For malaria diagnosis, thick and thin smears were prepared from finger-prick blood samples collected from patients, stained with Giemsa, and examined under a light microscope. Initial diagnosis was performed in the local hospital or clinics by field microscopists on duty. Confirmed malaria patients were treated by the hospital and clinic staff according to the local malaria treatment guidelines [8]. All blood smears were then shipped to a nearby project field laboratory and re-examined by two experienced microscopists to identify the species. When the results were discrepant, the slides were re-examined to obtain the final diagnosis.

The study protocol was reviewed and approved by the local Bureau of Health at Kachin and institutional review boards at Kunming Medical University, China Medical University, and Pennsylvania State University.

### Data analysis

Initial slide microscopy results of the hospitals and clinics were compared to those from the project laboratory using the Cohen’s kappa statistic and the McNemar’s test to evaluate if the discordance was differential. Cases of malaria were compared to febrile non-malaria cases using the χ^2^ test for categorical variables. For polytomous variables like occupation, education, and age categories, if there was an overall difference between the two groups, a reference group was chosen, and all subgroups were contrasted with this reference group. The Student’s *t* test was used for continuous variables when they were normally distributed or the Wilcoxon rank sum test for non-parametric analyses. Odds ratios were used to quantify the magnitude of association between the factors of interest and outcomes. To adjust for confounding, logistic regression was used to compute odds ratios after adjusting for the effects of age and gender. Test based methods were used to compute 95% confidence intervals (CI) and *P* values were interpreted in a two-tailed fashion. The Cochran–Armitage test was used to evaluate the linear trend of malaria burden over time. Seasonal index for a given month was calculated using the 6-year average number of cases observed in that particular month divided by the monthly-mean number of all cases over the six-year study period [9]. A value close to 1.0 indicates no significant shift from expectation for a particular month from the overall monthly mean [10]. Climate data were obtained from a nearby meteorological station in Yingjiang County, China, for the period between 2011 and 2016 (the highest and lowest monthly temperatures) and monthly precipitation for 2016.

## Results

### Demographics of suspected and confirmed malaria cases

During the six-year study period, a total of 31,326 febrile patients suspected of having malaria presented to the Laiza township hospital and nearby clinics in the northeastern Myanmar border. Malaria infections accounted for a substantial proportion of the febrile illnesses with 6175 (19.7%) confirmed slide-positive cases. Comparison of malaria diagnosis between field microscopists from the hospital and clinics and expert microscopists in the field laboratory revealed significant discrepancies (Table 1). The unweighted Cohen’s kappa was 0.61 (95% CI 0.57–0.65), suggesting that there was not a very good concordance between the diagnosis of the field and expert microscopists. A total of 1374 (22%) malaria cases were misdiagnosed as not being malaria in the field and hence did not receive anti-malarial treatment. Among the malaria cases, 20% of the *P. vivax* cases and 55% of the *P. falciparum* were misclassified in the field. Analyses of the malaria results presented are based on the diagnosis of the expert microscopists in the project laboratory.

**Table 1:** Comparison of malaria diagnosis between field microscopy at the treatment facilities and expert microscopy at the research laboratory

Field microscopy*	Expert microscopy*						
	Negative	Pf	Pv	Pm	Po	Mixed	Total
Negative	25,151	216	1107	13	2	36	26,525
Pf	0	215	17	0	0	2	234
Pv	0	39	4473	5	0	13	4530
Pm	0	0	9	3	0	0	12
Po	0	0	0	0	1	0	1
Mixed	0	11	1	0	0	12	24
Total	25,151	481	5607	21	3	63	31,326

Pf, *P. falciparum*; Pv, *P. vivax*; Pm, *P. malariae*; Po, *P. ovale*; mixed, mixed infection by *P. falciparum* and *P. vivax*

* Unweighted kappa (95% CI) 0.61 (0.57–0.65)

Comparisons between confirmed malaria cases and febrile non-malaria cases revealed significant differences in their demographic characteristics. There was a significantly higher proportion of males (62.8%) in the confirmed malaria cases compared to the febrile malaria-negative group (Table 2). The age structure was also significantly different for the two groups (*P* < 0.0001), with children 5–14 years and adults 15+ years of age being at a higher risk of malaria compared to children under 5 (Table 2). Compared to indoor workers, farmers, soldiers, and students were at a higher risk of malaria. Experiencing a previous episode of malaria within the last 12 months was also associated with a higher risk of clinical malaria (*P* < 0.0001).

**Table 2:** Demographic characteristics of confirmed malaria and non-malaria fever cases

Characteristics	Confirmed malaria, N (%)	Malaria negative fever, N (%)	*P* value*
All	6175	25,151	
Sex			
Male	3876 (62.8)	14,513 (57.7)	< 0.0001
Female	2299 (37.2)	10,638 (42.3)	
Age (year)			
Mean ± SD	18.9 ± 13.4	22.1 ± 16.6	< 0.0001
Median (Q1–Q3)	17 (9–24)	20 (7–31)	< 0.0001
< 5	573 (9.3)	4294 (17.1)	Reference
5–14	2023 (32.8)	5007 (19.9)	< 0.0001
15–24	2077 (33.6)	6003 (23.9)	< 0.0001
25+	1502 (24.3)	9847 (39.2)	< 0.05
Occupation			
Indoor worker^a^	891 (14.4)	5675 (22.6)	Reference
Farmer	889 (14.6)	4597 (18.3)	< 0.0001
Business person	19 (0.3)	135 (0.5)	ns
Manual labor^b^	113 (1.8)	706 (2.8)	ns
Soldier	850 (13.7)	3607 (14.3)	< 0.0001
Student	2872 (46.5)	6681 (26.6)	< 0.001
Other	541 (8.7)	3750 (14.9)	ns
Education^c^	N = 2976	N = 14,673	
Primary or less	497 (16.7)	2911 (19.8)	ns
Middle/high school	2400 (80.6)	11,478 (78.2)	
University or above	79 (2.7)	284 (1.9)	
Ethnicity			
Non-Kachin	264 (4.3)	1063 (4.2)	ns
Kachin	5911 (95.7)	24,088 (95.8)	
Malaria history^d^	N = 5534	N = 18,585	
Yes	115 (2.1)	126 (0.7)	< 0.0001
No	5419 (97.9)	18,459 (99.3)	

*ns* not significant

* Comparison between malaria positive and negative groups

^a^Indoor workers include office workers, housewives/housekeepers, and teachers

^b^Manual labor include factory/construction workers, lumberjack, plantation workers, temporary job/labor, hunters, miners, herdsman and gardeners/bush clearing

^c^Education: included only age 18 years and above

^d^Malaria history reported in the previous 12 months

### Seasonality and annual trend of clinical malaria incidence

**Fig. 1:**
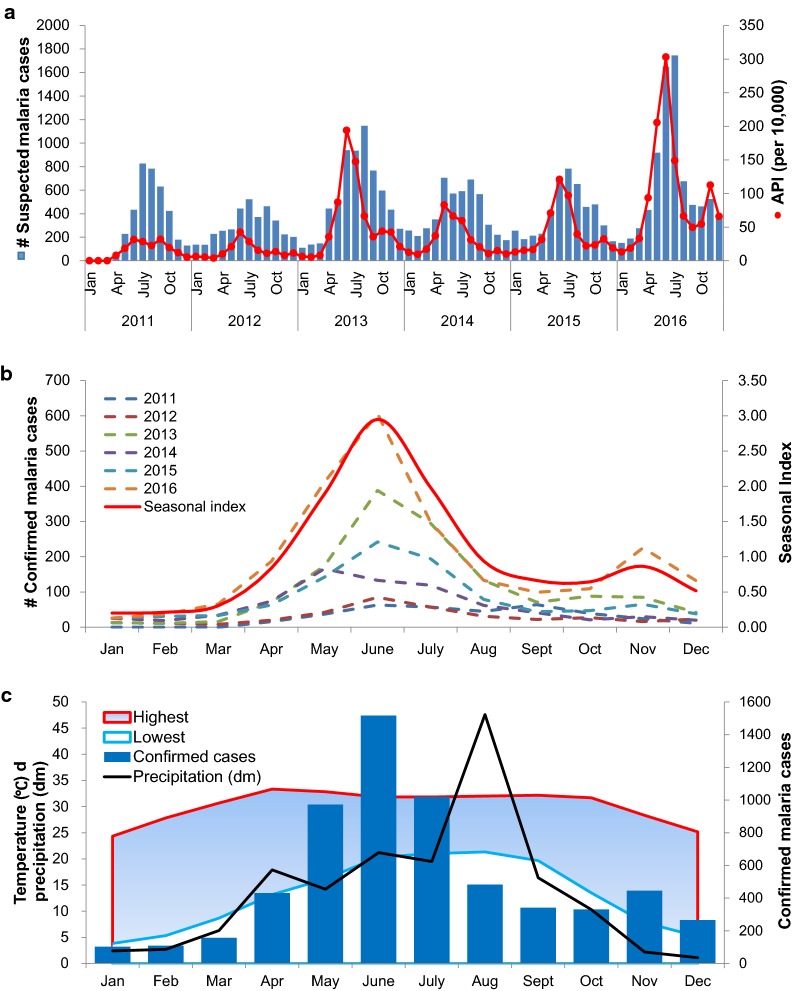
Trend and dynamics of malaria incidence in Laiza township at the China–Myanmar border during 2011–2016. **a** Dynamics of suspected malaria cases (blue bars) and annual parasite incidence (API, incidence number per 10,000 population, red dots). **b** Monthly confirmed malaria cases in different years (left y axis) and average seasonal index (right y axis) showing clear seasonality of malaria incidence. Note the major and minor peaks of malaria cases in June and November, respectively. Seasonal index = average number of cases for a respective month/monthly mean number of cases for all months during the 6-year study period. **c** Total monthly malaria cases (blue bars), precipitation in 2016 (black line) and average temperature (shadowed between the highest and lowest daily temperature)

Despite the region pursuing malaria elimination, there was no decrease in the annual incidence of malaria, with outbreaks occurring in 2013 and 2016 (Fig. 1a). *Plasmodium falciparum* and *P. vivax* cases had opposite trends over the study period (Table 3). Whereas *P. falciparum* cases showed a significant declining trend (*P* < 0.0001 for linear trend), there was a significant increase of *P. vivax* malaria over time (*P* < 0.0001 for linear trend) (Table 3). In addition, febrile illnesses showed a clear seasonal trend with most of the cases concentrated in the rainy season (May–September) (Fig. 1a). Similarly, malaria cases also displayed well-defined seasonality, peaking in May–July (Fig. 1b). The seasonal malaria index reached its highest level (2.95) in June. Interestingly, a minor peak of malaria cases was observed in November of 2013, 2015 and 2016 (Fig. 1a, b). Intensive malaria transmission in April–August coincided with the local rainy season and hot weather, whereas transmission was much lower in the dry and cooler months (Fig. 1c), which mimics the seasonal dynamics of the abundance of *Anopheles* mosquitoes [11, 12].

**Table 3:** Risk factors associated with *P. falciparum* and *P. vivax* slide positivity

Characteristic	Negative cases N (%)	*P. falciparum* cases		*P. vivax* cases	
		N (%)	AOR (95% CI)	N (%)	AOR (95% CI)
All	25,151	481 (7.8)		5607 (90.80)	
By year					
2011	3332 (13.3)	117 (24.3)	1^@^	209 (3.7)	1^@^
2012	3235 (12.9)	97 (20.17)	0.74 (0.57–0.98)*	240 (4.3)	1.21 (1.00–1.47)*
2013	5058 (20.1)	155 (32.22)	0.81 (0.63–1.04)	1218 (21.7)	3.99 (3.42–4.66)***
2014	4175 (16.6)	59 (12.27)	0.37 (0.27–0.51)***	682 (12.2)	2.73 (2.32–3.21)***
2015	3806 (15.1)	10 (2.08)	0.07 (0.04–0.13)***	993 (17.7)	4.28 (3.66–5.01)***
2016	5545 (22.1)	43 (8.94)	0.21 (0.15–0.30)***	2265 (40.4)	6.53 (5.63–7.57)***
Gender					
Female	10,638 (42.3)	121 (25.2)	1	2154 (38.4)	1
Male	14,513 (57.7)	360 (74.8)	2.20 (1.79–2.70)***	3453 (61.6)	1.16 (1.09–1.23)***
Age					
< 5	4294 (17.1)	18 (3.7)	1	550 (9.8)	1
5–14	5007 (19.9)	83 (17.3)	3.88 (2.33–6.47)***	1914 (34.1)	2.98 (2.69–3.30)***
15–24	6003 (23.9)	200 (41.6)	7.87 (4.85–12.77)***	1849 (33.0)	2.40 (2.16–2.66)***
25+	9847 (39.2)	180 (37.4)	4.43 (2.72–7.19)***	1294 (23.1)	1.03 (0.92–1.14)
Occupation^a^					
Indoor worker	5675 (22.6)	62 (12.9)	1	818 (14.6)	1
Business person	135 (0.54)	2 (0.42)	1.16 (0.28–4.81)	17 (0.30)	0.84 (0.50–1.39)
Manual labor	706 (2.8)	13 (2.70)	1.50 (0.82–2.76)	97 (1.7)	0.92 (0.74–1.16)
Farmer	4597 (18.3)	101 (21.0)	1.83 (1.32–2.54)**	768 (13.7)	1.15 (1.03–1.28)*
Soldier	3607 (14.3)	161 (33.5)	3.08 (2.23–4.24)***	675 (12.0)	1.19 (1.06–1.34)**
Student	6681 (26.6)	128 (26.6)	1.24 (0.89–1.74)	2711 (48.4)	2.38 (2.16–2.63)***
Other	3750 (14.9)	14 (2.9)	0.22 (0.12–0.41)***	521 (9.3)	0.77 (0.67–0.89)**
Education^b^					
Primary or less	2911 (19.8)	75 (36.2)	1	413 (47.6)	1
Middle/high school	11,478 (78.2)	245 (61.1)	0.63 (0.48–0.82)**	2118 (50.9)	1.06 (0.94–1.19)
University or above	284 (1.9)	11 (2.7)	1.08 (0.57–2.08)	67 (1.4)	1.31 (0.98–1.75)
Ethnicity					
Non-Kachin	1063 (4.2)	42 (8.7)	1	218 (3.9)	1
Kachin	24,088 (95.8)	439 (91.3)	0.51 (0.37–0.70)***	5389 (96.1)	1.00 (0.86–1.17)
Bednet use					
No	370 (1.5)	38 (7.9)	1	118 (2.1)	1
Yes	24,781 (98.5)	443 (92.1)	0.19 (0.14–0.27)****	5489 (97.9)	0.67 (0.54–0.82)**
Travel history					
No	25,069 (99.7)	462 (96.1)	1	5574 (99.4)	1
Yes	82 (0.33)	19 (4.0)	12.20 (7.31–20.36)***	33 (0.59)	1.80 (1.20–2.70)**
Malaria history^c^					
No	18,459 (99.3)	276 (94.5)	1	5101 (98.1)	1
Yes	126 (0.68)	16 (5.5)	8.35 (4.88–14.30)***	98 (1.9)	2.57 (1.97–3.35)***

*AOR* adjusted odds ratios, adjusted for age and gender

*, **, and *** Significant difference at *P *< 0.05, < 0.01, and < 0.0001 respectively, as determined by regression analysis

^@^ Cochrane–Armitage test for trend, *P *< 0.0001

^a^Same as in Table 2 for definition of indoor worker and manual labor

^b^Only included individuals of age 18 years and above

^c^Malaria reported in the past 12 months

### *Plasmodium vivax* as the predominant parasite species

**Fig. 2:**
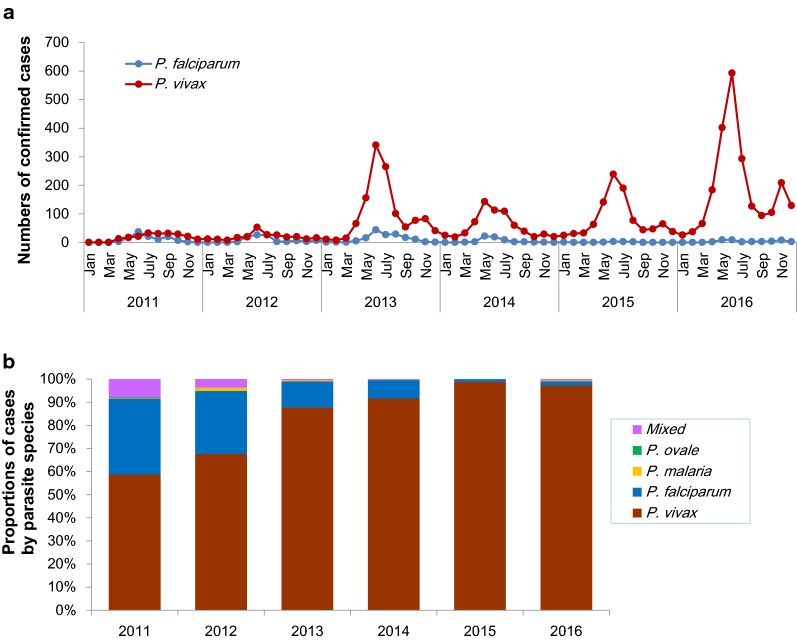
Dynamics of confirmed *P. vivax* and *P. falciparum* cases (**a**) and percentages of individual parasite species (**b**)

All four human malaria parasite species were detected in the study region. *Plasmodium vivax* was the predominant species and accounted for 5607 (90.80%) of the confirmed cases, followed by 481 (7.79%) cases of *P. falciparum*. There were 21 (0.34%) *Plasmodium malariae*, three (0.05%) *Plasmodium ovale* and 63 (1.02%) mixed infections. The rising trend of malaria may be attributed primarily to an increase of vivax malaria (Fig. 2a). In the last 6 years, vivax cases increased more than tenfold, from 209 in 2011 to 2265 in 2016. Of note, the two outbreaks in 2013 and 2016 were both due to *P. vivax*. In contrast, *P. falciparum* malaria cases decreased from 117 in 2011 to 43 in 2016. There were only ten *P. falciparum* cases in 2015.

### Risk factors for clinical malaria

Whereas males experienced a higher odds of vivax malaria [OR = 1.16 (95% CI 1.09–1.23)], this risk was more pronounced in males for falciparum malaria [OR = 2.20 (95% CI 1.79–2.70)] (Table 3). Similarly, ~ 80% of all falciparum malaria was experienced by older children and young adults (15+ years of age) in contrast to ~ 55% of vivax malaria in this age group. Compared to indoor workers, a low risk group for malaria, soldiers had more than three times the odds of falciparum malaria [OR = 3.08 (95% CI 2.23–4.24)], whereas this association was not as strong for vivax malaria [OR = 1.19 (95% CI 1.06–1.34)]. This species-related pattern was the opposite for students, who experienced higher odds for vivax malaria [OR = 2.38 (95% CI 2.16–2.63)] compared to falciparum malaria [OR = 1.24 (95% CI 0.89–1.74)]. While use of bed nets had a significant association with protection against vivax malaria [OR = 0.67 (95% CI 0.54–0.82)], this protective effect was more profound for falciparum malaria [OR = 0.19 (95% CI 0.14–0.27)].

A very small number of patients reported having traveled within the previous 2 weeks. A travel history was associated with increased odds of having malaria. In particular, those with travel histories had more than 12 times higher odds of falciparum malaria. Among the febrile patients, information about having a previous episode of malaria was recorded for 24,119 patients. A small proportion (1.0%) of these patients reported having malaria in the previous 12 months. Patients with malaria histories had almost eight- and three-times higher odds of acquiring falciparum malaria and vivax malaria, respectively, compared to those without a history of malaria.

### Clinical signs and symptoms of falciparum and vivax malaria

Most of the patients presented for treatment with a history of 2–3 days of fever. About 9% had more than 4 days of fever before seeking medical treatment. A higher proportion of patients with falciparum malaria experienced loss of appetite, dizziness, joint pain, and difficulty breathing, compared to febrile non-malaria patients. In contrast, all these symptoms were significantly lower among vivax patients compared to febrile non-malaria patients. Comparing the symptoms for the two parasite species, a significantly higher proportion of vivax patients had shivering/chills than falciparum malaria patients, whereas falciparum malaria patients reported significantly higher proportions of diarrhea, abdominal pains, nausea, stomachache, loss of appetite, dizziness, coughing, joint pain, and difficulty breathing (*P* < 0.0001; Table 4).

**Table 4:** Comparison of clinical signs and severe symptoms in *P. falciparum*, *P. vivax* and febrile negative cases

Features	Negative (N = 25,151)	*P. falciparum* (N = 481)		*P. vivax* (N = 5607)		*P* value
	N (%)	N (%)	*P* value*	N (%)	*P* value^#^	(*Pf* vs *Pv*)
With fever history	23,510 (93.5)	445 (92.5)		5370 (95.8)		
Days of fever						
Mean ± SD	2.27 ± 1.64	2.85 ± 1.58		2.43 ± 1.34		< 0.0001^a^
Median (Q1–Q3)	2 (2–3)	3 (2–3)		2 (2–3)		
Clinical signs						
Vomiting	1081 (4.3)	19 (4.0)	ns	142 (2.5)	< 0.0001	ns
Diarrhea	288 (1.2)	12 (2.5)	< 0.01	23 (0.4)	< 0.0001	< 0.0001
Abdominal pains	302 (1.2)	11 (2.3)	< 0.05	27 (0.5)	< 0.0001	< 0.0001
Nausea	207 (0.8)	16 (3.3)	< 0.0001	24 (0.4)	< 0.001	< 0.0001
Headache	10,021 (39.8)	170 (35.3)	< 0.05	2257 (40.3)	ns	< 0.05
Stomach ache	384 (1.5)	18 (3.7)	< 0.0001	31 (0.6)	< 0.0001	< 0.0001
Loss of appetite	1059 (4.2)	52 (10.8)	< 0.0001	148 (2.6)	< 0.0001	< 0.0001
Dizziness	1540 (6.1)	42 (8.7)	< 0.01	166 (3.0)	< 0.0001	< 0.0001
Coughing	1556 (6.2)	35 (7.3)	ns	110 (2.0)	< 0.0001	< 0.0001
Joint pain	2429 (9.7)	74 (15.4)	< 0.0001	342 (6.1)	< 0.0001	< 0.0001
Difficulty breathing	1204 (4.8)	65 (13.5)	< 0.0001	174 (3.1)	< 0.0001	< 0.0001
Shivering/chills	5293 (21.0)	106 (22.0)	ns	2556 (45.6)	< 0.0001	< 0.0001
Severe symptoms^b^	N = 3444 (13.7)	N = 128 (26.6)		N = 297 (5.3)		
Coma	27 (0.78)	0	ns	3 (1.0)	ns	ns
Convulsions	2712 (79)	87 (68)	< 0.0001	173 (58)	< 0.0001	< 0.0001
Respiratory distress	1180 (34)	65 (51)	< 0.0001	166 (56)	< 0.0001	< 0.0001
Acute pulmonary edema	26 (0.75)	0	ns	4 (1.3)	ns	ns
Renal failure	2 (0.06)	0	< 0.05	1 (0.33)	ns	ns
Jaundice	4 (0.11)	3 (2.3)	< 0.0001	5 (1.7)	ns	< 0.05
Severe anemia	37 (1.1)	15 (12)	< 0.0001	9 (3.0)	ns	< 0.0001
Other	39 (1.1)	1 (0.78)	ns	6 (2.0)	ns	ns

*ns* not significant

* Comparison between *P. falciparum* cases and febrile, malaria-negative fever cases

^#^ Comparison between *P. vivax* cases and febrile, malaria-negative fever cases

^a^ Mann–Whitney test

^b^Column percentages for symptoms of severity are percent of all severe cases

Both falciparum and vivax malaria patients had presentations with severe disease symptoms (Table 4). The proportion of patients with severe falciparum malaria (26.6%) was higher than that with severe vivax malaria (5.3%). In addition, of the vivax patients, three had coma, two had acute pulmonary oedema, and one suffered renal failure. The most common severe symptoms for both *P. falciparum* and *P. vivax* malaria included convulsions and respiratory distress, although their proportions were significantly higher in the severe falciparum patients. In addition, severe falciparum malaria cases had a significantly higher proportion (3.12%) of presentations with severe anaemia than severe vivax malaria patients (0.16%) (*P* < 0.0001).

## Discussion

As malaria control along porous international borders is critical to achieve the goal of malaria elimination, this study conducted surveillance in a malaria-endemic border region with high-risk mobile populations. Consistent with the WHO reports on rising trends of malaria cases for years 2016 and 2017, the China–Myanmar border area also showed a worrisome increase in the burden of malaria with vivax malaria accounting for most of the clinical cases.

Despite intensified malaria control efforts with large bed net coverage and availability of appropriate recommended anti-malarial treatment, the annual malaria incidence for vivax malaria steadily increased over the 6 years of the study (2011–2016). While the cause for this increase is not obvious, population genetic analysis from the Laiza area between 2011 and 2013 revealed reduced genotypic evenness of the parasite population [13], suggesting that the expansion of certain parasite genotypes may be responsible for the outbreaks.

Alternatively, drug resistance may also be partially responsible, as the efficacy chloroquine in the treatment of vivax malaria in this region was shown to be deteriorating [14]. Other contributing factors to this rising trend of vivax malaria could be the significant increase in *Anopheles* vector density, as observed in 2013 [12].

Increased risk of malaria in school-aged children and their potential to be a reservoir of transmission has been observed in many parts of the world [15–17]. Consistent with these and other findings from the area [8, 18], students accounted for the largest proportion (47%) of clinical malaria cases. Though not fully understood, one reason for the age bias could be related to the developing immunity against clinical malaria in this age group. Soldiers were another high-risk group in the area and accounted for approximately 14% of all malaria cases. Since soldiers constitute a very small proportion of the total population, this would translate to a very high incidence rate in this group. Most border malaria surveillance reports focus on migrant workers and mobile populations. This study shows that soldiers who patrol border and forested areas, especially in unstable areas, may be an important source of malaria transmission and control efforts should target this group for interventions. Strikingly, soldiers also accounted for about 34% of all falciparum malaria cases, suggesting that they might be an important source for low-level maintenance of falciparum malaria in the region.

People with a travel history in the study area also carried significantly increased odds of having acute malaria infections. On the China side, adult men and travel history to Myanmar were major risk factors for malaria acquisition, which highlights the contribution of human cross-border movement as an important source of malaria introduction [19]. This one-directional cross-border movement of parasites was further implied from genetic evidence, which showed that migration of the parasites from the neighbouring sites in Myanmar to Yunnan in China was asymmetrical [20]. To realize the regional goal of malaria elimination and prevent parasite re-introduction, heightened border inspection on migrant population is necessary, as removal of the last transmission foci on both sides of the border is critical.

Falciparum and vivax malaria differed significantly in their clinical symptoms and characteristics. The large difference in the odds ratios for protection from bed net use (0.19 vs 0.67) and risk with travel history (12.2 vs 1.8) might suggest that a large fraction of the vivax malaria might be due to relapses, since bed nets should be equally effective in protection against exposure to *Anopheles* mosquitoes carrying *P. vivax or P. falciparum*. Vivax malaria is often described as ‘benign tertian’ malaria. In our study, approximately 300 vivax patients presented with symptoms of severe malaria, including coma, febrile convulsions, respiratory distress, acute pulmonary edema, renal failure and severe anemia, further emphasizing the need for improved clinical management of this parasite.

An important finding from the PCD study is that field diagnosis missed a substantial number (22%) of malaria cases. As a result, more than a fifth of the cases did not receive antimalarial treatment. Surveillance data from routine surveillance hence significantly under-estimates the true burden of malaria. Because 8% of falciparum cases was wrongly classified as vivax, these *P. falciparum* cases received inappropriate treatment with chloroquine and could have been treatment failures because of chloroquine resistance in the area. This could also be a contributing factor to the low-level persistence of falciparum malaria observed throughout the study period. Strengthened training of field microscopists and the deployment of ultrasensitive rapid diagnosis tests are needed to improve the diagnostic accuracy.

## Conclusions

Malaria incidence in the China–Myanmar border area has experienced an overall upward trend in recent years, amidst the continuous decline of malaria cases in the entire GMS. *Plasmodium vivax* has become a major obstacle for malaria elimination, and it was responsible for an increasing proportion of the clinical malaria incidence as well as the two outbreaks. Thus, improved case management and more effective treatment of vivax malaria are needed, especially with the evidence of reduced efficacy of chloroquine-primaquine for the treatment of vivax malaria. In addition, the presence of a substantial number of cases missed or misdiagnosed in the field highlights the need for strengthened training of field microscopists and deployment of more reliable diagnostic methods. Targeted control measures need to focus on vulnerable populations such as soldiers, children, and migrants. Altogether, this study highlights the need for effective strategies to shrink the large parasite reservoir in this region. Elimination of border malaria is also essential to avoid malaria re-introduction into neighbouring countries, and to achieve the goal of regional malaria elimination.

## Data Availability

All relevant data and materials are included in the manuscript.

## Abbreviations

GMS: Greater Mekong Subregion
IDP: internally displaced people
OR: odds ratio
PCD: passive case detection
